# Acute Case of Poisoning by Arsenite of Copper

**Published:** 1880-10

**Authors:** N. F. Thompson


AN ACUTE CASE OF POISONING BY ARSENITE OF COPPER (Paris Green).—A brown mare, 15½ hands high, 16 years old, was brought to the hospital, August 18th, for medical treatment.

HISTORY.—The previous night the horse had become loose in her stall, had eaten a quantity of Paris green and wheat that had been mixed for killing potato bugs, and which, by some oversight, had been carelessly left in the stable. It was not noticed that the mare had eaten the poison until late the next morning, and as no unusual symptoms were present, the animal was harnessed and driven to New York with a load of produce. On the road into the city, the owner noticed that the mare showed signs of weakness, was growing sluggish, and acting in an unusual manner. On being brought to the Infirmary I examined her, and found the visible mucus membranes highly injected; pulse 65 to the minute, small and wiry. Respiration increased in frequency and labored. Intervals of dulness were succeeded by colicky pains, and the extremities were cold. Considerable violent purging; abdomen fumid and tender, together with the general symptoms of intestinal irritation. The patient had the appearance of an animal suffering great pain. The thirst was considerable.

Diagnosis—Acute arsenical poisoning.

Treatment—Ordered the patient placed in a large box stall, and immediately administered the usual antidote:

Ferri Oxidum Hydratum, *quantum sufficit.*

Three ounces every ten minutes, to be followed by hydrated sulphate of magnesia,

8 drachms. The patient was comparatively easy for about two hours, when the colicky symptoms returned with increased violence; pulse ran up to 80 and 100 beats per minute, when she began to struggle violently, and died after a short time in intense agony.

Necropsy—10 hours after death.

On opening the abdominal cavity, there was an escape of a considerable quantity of fetid gas. The stomach was flaccid and soft; the gastric mucus membrane thickened and swollen, and covered with patches of a reddish-brown color. No perforations or ulcerations.

The same condition was found the whole length of the intestinal tract, being less marked as the distance from the stomach increased.

No urine was found in the bladder, the kidneys being markedly congested, as well as the liver and lungs.

N. F. Thompson, D. V. S.

